# TH17-phenotypes of juvenile idiopathic arthritis

**DOI:** 10.1186/1546-0096-12-S1-P29

**Published:** 2014-09-17

**Authors:** Inga Z Turtsevich, Gennadiy A Novik, Natalia V Bychkova

**Affiliations:** 1 Saint-Petersburg State Pediatric Medical University, Saint-Petersburg, Russian Federation; 2 The Federal State Budgetary Institute «The Nikiforov Russian Center of Emergency and Radiation Medicine» The Ministry of Russian Federation for Civil Defense, Emergencies and Elimination of Consequences of Natural Disasters, Saint-Petersburg, Russian Federation

## Introduction

Juvenile idiopathic arthritis (JIA) is a chronic disorder with unknown etiology and characterized by autoimmunity, infiltration of synovium by activated proinflammatory cells, synovial hyperplasia and progressive destruction of cartilage and bone.

IL-17A is a proinflammatory cytokine that is expressed in the inflamed synovium. Th17 cells have been identified as main producers of this cytokine. IL-17A is a potent inducer of various cytokines and chemokines. In addition, this cytokine has been shown to have additive or even synergistic effects with TNFα and IL-1β on cytokine induction and tissue destruction.

## Objectives

To characterize Th17 pathway in children with different subtypes of JIA as a mechanism for determining the nature of the clinical course and outcome of the disease.

## Methods

PB samples were obtained from 100 patients with different subtypes of JIA and 20 PB samples from healthy control. The bases of quantitative evaluation of Th17 cells were taken directly determination of CCR6 in PBMC by flow cytometry. Cytokines, such as IL-1β, IL-6, IL-17A and TNFα were determined in serum samples of the patients by ELISA.

## Results

Highest level of Th17 cells, IL-17A, IL-1β and IL-6 were detected in PB in children with «active» HLA B27-associated arthritis (p=0,001, p=0,001, p=0,007 and p=0,05, respectively) and systemic onset of JIA (p=0,002, p=0,021, p=0,04 and p=0,03, respectively) in compare with oligo-, polyarthritis where level of these parameters were lower or not significantly different from healthy control. Statistically significant differences in level of TNFα in serum in all subtypes of JIA were not obtained.

Level of IL-17A˃1,04 pg/ml and Th17 memory cells˃3,2% were associated with high risk of active disease (OR=3,727, p=0,003) and level of IL-17A˃1,04 pg/ml and IL-6˃10,1 pg/ml were associated with the risk of osteoporosis (OR=2,905, p=0,008).

In order to identify phenotypes of JIA hierarchical cluster analysis followed by discriminant analysis were used. Thus, can be distinguished at least Th17-dependant and Th17-independent phenotypes. Th17-dependant phenotype includes children with different subtypes of JIA with high disease activity and high risk of bone and cartilage destruction. Key inflammatory mediator of this phenotype is IL-17A. Th17-independent phenotype characterized by lower disease activity and lower risk of bone and cartilage destruction.

## Conclusion

Our data suggest that Th17 cells and their cytokines play a crucial role in pathogenesis of HLA B27-associated arthritis and systemic onset of JIA. This research allows determining the level of Th17 cells and IL-17A as markers of high risk of persistence of «active» disease, while low level of Th17 cells and IL-17A increase the chance of a favorable disease outcome.

## Disclosure of interest

None declared.

